# Lack of Association Between *qacE* and *qacE∆1* Gene Variants and Sodium Hypochlorite Resistance in Clinical Isolates of ESBL- and Carbapenemase-Producing *Klebsiella* spp. and *Enterobacter* spp., from Gaborone, Botswana

**DOI:** 10.3390/antibiotics14070662

**Published:** 2025-06-30

**Authors:** Pearl Ntshonga, Tlhalefo Dudu Ntereke, Tshiamo Zankere, Daniel Paul Morse, Garesego Koto, Irene Gobe, Giacomo Maria Paganotti

**Affiliations:** 1 School of Allied Health Professions, Faculty of Health Sciences, University of Botswana, Gaborone Pvt Bag 0022, Botswana; 201702229@ub.ac.bw (P.N.); kotog@ub.ac.bw (G.K.); gobei@ub.ac.bw (I.G.); 2 Botswana-University of Pennsylvania Partnership, Gaborone P.O. Box 45498, Botswana; ntereket@bup.org.bw (T.D.N.); zankeret@bup.org.bw (T.Z.); 3 Chemistry Department, United States Naval Academy, Annapolis, MD 21402, USA; morse@usna.edu; 4 Division of Infectious Diseases, Perelman School of Medicine, University of Pennsylvania, Philadelphia, PA 19104, USA

**Keywords:** class 1 integrons, *Enterobacter*, *Klebsiella*, *qacE*, sodium hypochlorite

## Abstract

**Background**: The *qacE* gene and its variant, *qacE∆1*, have been associated with resistance to antimicrobials and biocides. This poses a threat to infection prevention, control and treatment. Several studies investigated this relationship with conflicting results. The aim of this research was to determine the prevalence of *qacE* and *qacE∆1* in clinical extended spectrum β-lactamase- (ESBL) and carbapenemase-producing *Klebsiella* spp. and *Enterobacter* spp. and elucidate the association of these genes with resistance to sodium hypochlorite. **Methods**: This study included 216 isolates of ESBL- and carbapenemase-producing multidrug-resistant (MDR) *Klebsiella* spp. and *Enterobacter* spp. These isolates were identified by VITEK-2 analyser. The MIC and MBC of sodium hypochlorite were determined using the microbroth serial-dilution method. PCR was used to detect gene variants. A regression analysis investigated any association between *qacE* genotypes, MIC and MBC, as well as antimicrobial drug resistance profiles. **Results**: Overall, there was a high prevalence of *qacE* and *qacE∆1* variants (84.7%; 95% CI, 79.2–89.2). There was a high prevalence of *qacE∆1* (80.6%; 95% CI, 74.6–85.6) as compared to *qacE* (15.3%, 95% CI, 10.8–20.8). The MIC_50_ and MIC_90_ of the isolates ranged between 7031 mg/L and 9375 mg/L and 14,060 mg/L and 18,750 mg/L, respectively, while the MBC ranged from 48,750 mg/L to 18,750 mg/L. There was no association between *qacE* genotypes and high MIC and MBC as well as antimicrobial drug resistance. **Conclusions**: The MIC and MBC of sodium hypochlorite are higher than what is currently used for disinfection in Botswana. There is a high prevalence of *qacE* and *qacE∆1*; however, these genes do not seem to be associated with resistance to sodium hypochlorite.

## 1. Introduction

*Klebsiella* spp. and *Enterobacter* spp. are opportunistic nosocomial infection-causing bacteria that have rapidly gained notoriety and are included in the critical category of the World Health Organization’s (WHO) list of priority pathogens for which new antibiotics are urgently needed [1,2]. These bacteria cause a wide range of nosocomial infections [2,3]. Since both *K. pneumoniae* and *Enterobacter* spp. show extensive intrinsic and acquired resistance [4], treatment options are severely limited in infections caused by these bacteria, often leading to the use of last-resort antimicrobial drugs such as carbapenems and colistin [5].

Currently, β-lactam drugs are the most used and are composed of four groups: penicillins, cephalosporins, monobactams and carbapenems [6]. There are three main ways in which resistance to β-lactam drugs is achieved: enzymatic degradation, overexpression of efflux pumps and porin modification [6]. However, enzymatic degradation is a key method, with the production of ampC β-lactamases, cephalosporinases, ESBLs and carbapenemases [7]. Genetic elements such as integrons, plasmids and transposons play a critical role in arranging, expressing and spreading genes associated with resistance to β-lactam drugs. In fact, structures such as integrons also capture biocide resistance genes, and may drive co-selection for biocide/β-lactam resistant bacteria in the hospital environment [8].

Antimicrobial resistance (AMR) and antimicrobial drug resistant-associated mortality is disproportionately higher in low–middle income settings [9] such as Botswana, in which AMR is estimated to be among the top five leading causes of death [10]. It has also been reported that drug-resistant *K. pneumoniae* accounts for 24% of all fatal infections in this country [10]. In a 2019 global statistics report, it has been shown that 600,000 and 200,000 deaths were associated with multidrug-resistant (MDR) *K. pneumoniae* and *Enterobacter* spp., respectively [10,11]. The incidence of nosocomial infections in this region ranges between 2% and 49% with 3% to 11% of the cases resulting in fatalities [12]. These statistics can be attributed to several factors including lack of effective antibiotic policies, poor healthcare systems and inadequate infection prevention and control (IPC) practices [12,13].

The burden of *K. pneumoniae* and *Enterobacter* spp. infections in Botswana was clearly reflected in a 2017 study performed by Gezmu et al. [14], in which *K. pneumoniae* accounted for two-thirds of neonatal blood stream infections (BSIs), 80% of which exhibited the ESBL phenotype in a tertiary hospital in Botswana. Additionally, in a study by Mannathoko et al. [15], *K. pneumoniae* and *Enterobacter* spp. were the second and the third most common colonizers exhibiting resistance to both carbapenems and extended-spectrum cephalosporins, respectively. The reported study also demonstrated that colonization with extended-spectrum cephalosporins-resistant Enterobacterales (ESCrE) and carbapenem-resistant Enterobacterales (CRE) was higher in clinical as opposed to community settings [15].

The intricate relationship between MDR phenotype and the ability to persist and cause nosocomial infections is significant and requires a multifaceted, coordinated response to contain it. In this instance, effective IPC is essential, as the selection and spread of MDR can be driven by sub-standard IPC practices.

The spread of resistance in bacteria is attributed to their ability to acquire and share plasmids, transposons and, more importantly, integrons, which play a role in transferring and assembling genes associated with antimicrobial drug resistance [16]. Integrons are genetic elements that, although unable to move themselves, contain gene cassettes that can be mobilized to other integrons or to secondary sites in the bacterial genome, thereby spreading antibiotic resistance genes [16,17]. For example, the characterization of a Class 1 integron in clinical isolates of *E. cloacae* discovered the gene arrays *aac(6′)-Ib-cr5-arr-3-dfrA27* and *aac(6′)-Ib-catB8-aadA1*, which encode resistance for trimethoprim, aminoglycosides and chloramphenicol [18]. Class 1 integrons are the most abundant and clinically significant and serve as a good indicator for environmental pollution as they include genes that are captured and expressed in response to environmental stressors such as antibiotic and biocide pressure and thereby confer resistance to both antimicrobial drugs and biocides [16]. Class 1 integrons are composed of two conserved regions at the 3′- and 5′-ends, with a central variable region containing several resistance determinants sequentially inserted as cassettes. One of the genes harboured within these integrons is the quaternary ammonium compounds resistance determinant, *qacE*, and its active attenuated variant, *qacE∆1*, which belong to the Small Multidrug Resistance (SMR) family [16]. This gene encodes multi-substrate efflux pumps which confer adaptive response to antimicrobial drugs and biocides [16,19,20,21] in synergy with other resistance mechanisms, all together modulating the response of the bacteria to biocides and/or antibiotics [16]. Over the years several studies have sought to establish the relationship between *qacE* genotypes and antimicrobial drug resistance as well as antiseptic/biocide tolerance/resistance and have arrived at conflicting findings [19,22,23,24,25,26], as summarized by Ntshonga et al. [27].

The aim of this study was to explore the possible association of *qacE* and *qacE∆1* with biocide resistance in clinical isolates of *Klebsiella* spp. and *Enterobacter* spp., resistant to cephalosporins and carbapenem drugs. In particular, the study sought to determine the pattern of sodium hypochlorite (NaOCl) resistance, as the most used disinfectant in low-resourced *hospitals* in African countries, including Botswana. Sodium hypochlorite is used for the disinfection of benches and frequently contacted surfaces, as well as biological spills in Botswana hospitals, according to international guidelines. It is recommended to be used at 0.5% (5000 mg/L) for non-porous surfaces at a minimum contact time of 10 min. Alternatively, Cidex OPA, which belongs to the orthophtaldehyde group can be used, both being paired with 70% alcohol. Although hypochlorous acid, which is the active form of sodium hypochlorite, is not transported through an electrochemical proton gradient, which is the mechanism of action of the *qacE*/*qacE∆1* efflux pump, it induces a stress response which upregulates several bacterial physiologic processes including an increase in the frequency of horizontal gene transfer [28].

In the hospital environment, prolonged, non-discriminated use and sub-inhibitory exposure to NaOCl may drive the acquisition of Class 1 integrons and consequently, biocide and antimicrobial drug resistance [28]. Here we hypothesize that *qacE*/*qacE∆1* bacterial genes have been selected by prolonged exposure, and they are present at high frequency in Botswana.

## 2. Results

### 2.1. Selection of Bacterial Strains, Identification, and Antimicrobial Susceptibility Testing

**Figure 1:**
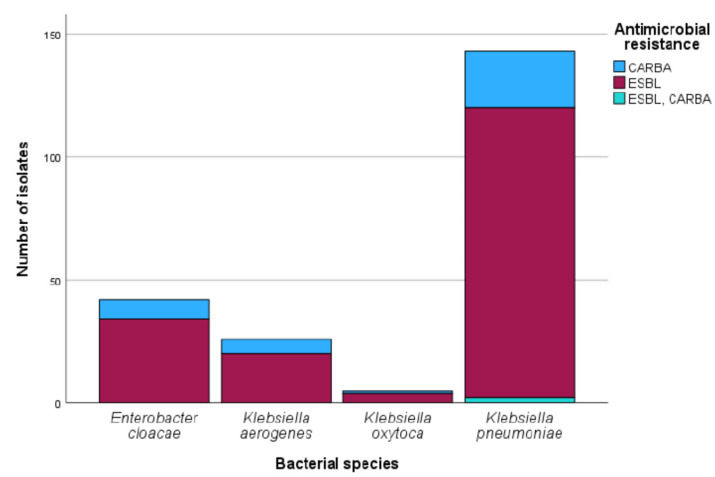
Antimicrobial resistance profile by bacterial species.

**Figure 2:**
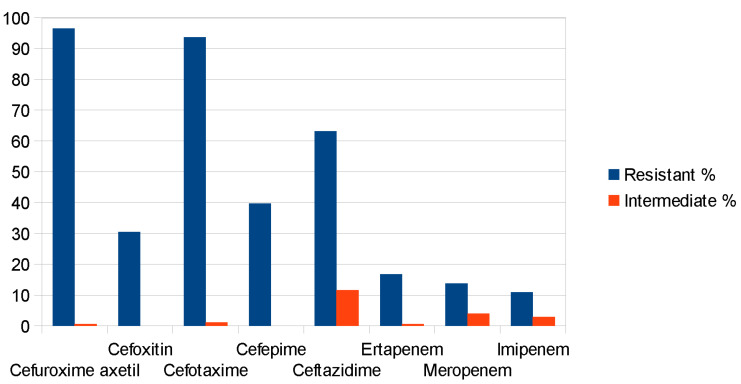
Resistance to cephalosporins and carbapenem molecules in *Klebsiella* spp.

**Figure 3:**
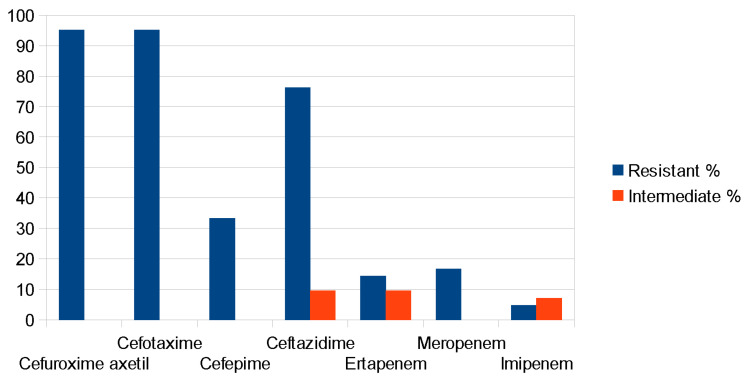
Resistance to cephalosporins and carbapenem molecules in *Enterobacter* spp. Note that cefoxitin resistance is not reported since *Enterobacter* spp. shows intrinsic resistance to it due to chromosomal AmpC β-lactamase.

One hundred forty-eight (148) and sixty-eight multidrug-resistant *Klebsiella* and *Enterobacter* spp. isolates, respectively, were initially selected and evaluated by the VITEK-2. The overall 216 isolates were speciated as *K. aerogenes* (26), *E. cloacae* (42), *K. oxytoca* (5) and *K. pneumoniae* (143), which showed resistance to β-lactam antibiotics and carbapenems (ESBL and CARBA, in Figure 1). The isolates were resistant to cefuroxime axetil (96.5%), cefotaxime (93.7%), ceftazidime (63.2%), cefoxitin (30.5%), cefepime (39.7%), ertapenem (16.7%), meropenem (13.8%) and imipenem (10.9%) for *Klebsiella* spp. and cefuroxime axetil (95.2%), cefotaxime (95.2%), ceftazidime (76.2%), cefepime (33.3%), ertapenem (14.3%), meropenem (16.7%) and imipenem (4.8%) for *Enterobacter* spp. (see Figure 2 and Figure 3 and Supplementary Table S1).

### 2.2. MIC and MBC of Sodium Hypochlorite

Data presented in Table 1 summarize the MICs required to inhibit 50% (MIC_50_) and 90% (MIC_90_) of the bacterial population, along with the mean minimum bactericidal concentration of sodium hypochlorite against different *Klebsiella* and *Enterobacter* species. Although both MIC and MBC values are higher than 5000 mg/L, they are comparable across all bacterial isolates ranging between 7031 mg/L and 9375 mg/L, 14,060 mg/L and 18,750 mg/L and 48,750 mg/L and 56,250 mg/L for the MIC_50_, MIC_90_ and Mean MIC, respectively. The comparison of MIC_50_ of sodium hypochlorite in isolates carrying [*qacE* and/or *qacEΔ1*] vs. [no gene], using the Mann–Whitney U test, did not show statistically significant differences (*p* = 0.4354 for MIC_50_). In addition, the comparison of mean MBC of sodium hypochlorite in isolates carrying [*qacE* and/or *qacEΔ1*] vs. [no gene], using the Student’s *t*-Test, did not show statistically significant differences (*p* = 0.5097). Finally, binary logistic regression analysis was performed to compare the ESBL and CARBA (ESBL+CARBA were excluded) phenotypes distribution according to the *qacE* genotypes and bacterial species. No significance was recorded (*p* = 0.560 and *p* = 0.447, for *qacE* genotypes and bacterial species, respectively).

### 2.3. PCR and Restriction Digest

**Figure 4:**
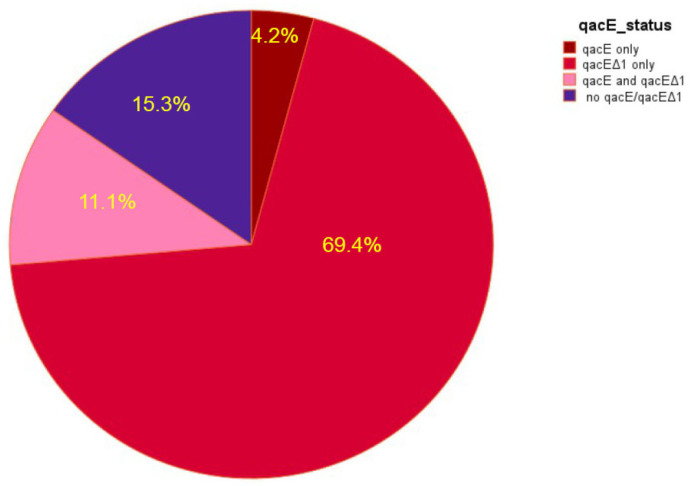
Proportion of isolates carrying *qacE*, *qacEΔ1* and no gene.

Results from the molecular analysis showed that there was an overall 84.7% of isolates carrying the *qacE* gene and/or its variant *qacEΔ1* (Figure 4). Frequencies by species of the two variants of the gene are shown in Table 2.

**Figure 5:**
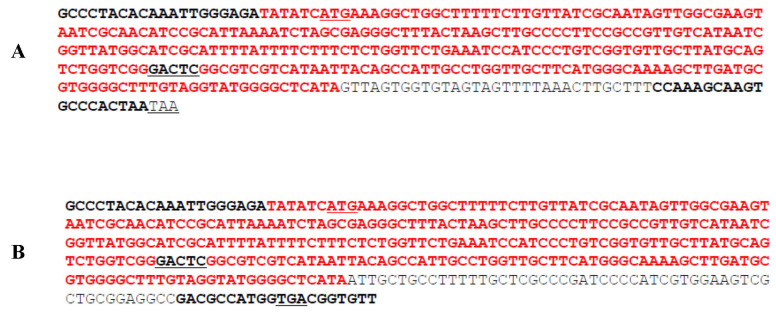
(**A**): *qacE* PCR product is 359 bp; *HinF1* fragments are 220 bp and 139 bp; (**B**): q*acE∆1* PCR product is 381 bp; *HinfI* fragments are 220 bp and 161 bp. Priming sites are in bold; *HinfI* site is in bold and underlined; start (ATG) and stop (TAA for *qacE* and TGA for *qacE∆1*) codons are underlined; red (and upstream primer) sequences are the same in the two genes.

**Figure 6:**
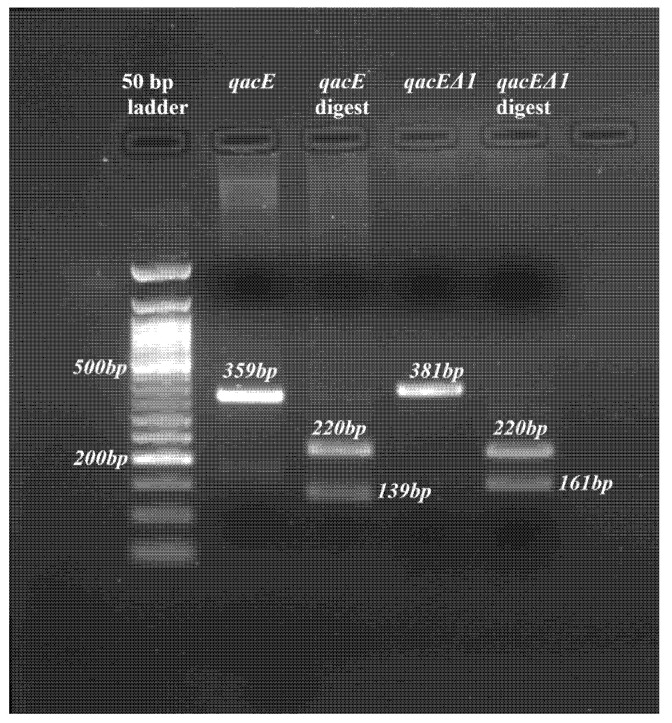
Representative *qacE* and *qacE∆1* PCR products along with the corresponding *Hinf1* digestion products.

Furthermore, digestion with the *HinfI* restriction enzyme was performed. As expected, digestion with *HinfI* produced 221 bp and 139 bp for the wildtype gene and 221 and 161 bp for the deletion variant, confirming the previewed sizes of the PCR fragments (Figure 5 and Figure 6).

## 3. Discussion

The containment of nosocomial infections relies on the continued effectiveness of the chemicals that are used to control the bioburden in the hospital environment. However, sub-inhibitory exposure may drive the development and selection of biocide tolerant/resistant strains as well as induce cross-resistance to antibiotics, usually driven by Class 1 integrons [27,29]. Resistance in problematic nosocomial pathogens is usually driven by Class 1 integrons which carry genes associated with AMR and genes associated with biocide resistance, including *qacE* and its variant *qacEΔ1* [16].

For this study, we adopted the gene sequences for *qacE* and its attenuated variant from Kazama et al. [22] to identify priming sites, coding regions and start and stop codons of the *qacE* gene and its variant *qacE∆1* (Figure 5). Our PCR amplification yielded products of expected band sizes, specifically 359 bp for *qacE* and 381 bp for *qacE∆1* (Figure 6). Gel electrophoresis results were congruent with theoretical predictions based on *HinfI* digestion. Our findings reconcile discrepancies noted in the literature regarding primer sequences and expected product sizes for *qacE* and *qacE∆1*. For instance, Kazama et al. [22] and Kücken et al. [23] used different primers for detecting *qacE* in *E. cloacae*. Similarly, variations were observed in the primer sequences used by Kazama et al. [22] and Hadadi et al. [30] for *qacE* and *qacE∆1* in *E. coli*. However, there seems to be a consensus regarding the primer sequences for *qacE* and *qacE∆1* in *K. pneumoniae*, which have been widely accepted and used, for example, by Guo et al. [31] and Vijayakumar et al. [25]. By adopting established primer sequences and confirming their utility through experimental validation, we demonstrated the reproducibility of this molecular detection method for *qacE* and *qacE∆1*. The primer sequences from Kazama et al. [22] were effective in amplifying *qacE* and *qacE∆1* genes in *Klebsiella* spp. and *Enterobacter* spp.

Similarly to Abuzaid et al. [24] and Liu et al. [32], we found a high prevalence of *qacEΔ1* variant in clinical isolates of ESBL-producing and carbapenemase-producing bacteria species as compared to *qacE*, with an overall 84.7% of isolates carrying either *qacE* only, *qacEΔ1* only or both variants of the gene (Table 2, Figure 2). As *qacEΔ1* constitutes the 3′ conserved segment of Class 1 integrons and can therefore be used as a proxy for the prevalence of these elements [23], our results indicate a high prevalence of these integrons. Furthermore, isolates that carry both *qacE* and *qacEΔ1* also carry two types of Class 1 integrons: ones that have *qacE* in their 3′ conserved segment and ones that contain *qacEΔ1* in that segment [22].

In this study, there was no significant association between the distribution of *qacE* genotypes and either ESBL-producing or carbapenemase-producing phenotypes (binary logistic regression analysis, *p* = 0.560). This is similar to Abuzaid et al. [24] and Vijayakumar et al. [25], who reported no significant association of *qacE* and/or *qacEΔ1* with AMR. However, this contradicts findings by Wang et al. [33], who characterized the composition of Class 1 integrons in *K. pneumoniae* and subsequently the association of Class 1 integrons with resistance to β-lactam drugs, and Kücken et al. [23], who associated *qacEΔ1* with resistance to non-beta lactam drugs in *E. cloacae*. We hypothesize that the *qacE* gene and *qacEΔ1* are part of a panel of genes associated with antimicrobial resistance, where multiple genes operate synergistically to confer resistance. This model has already been described for *K. pneumoniae* for other SMR genes [34]. Consequently, attributing the resistant phenotype solely to the presence of one member of this panel may not always show an association.

Although *qacE* and its attenuated variant have been associated with resistance/tolerance to several biocides, there was no statistically significant difference in the MIC_50_ and mean MBC of NaOCl between the group of isolates that harboured either *qacE*, *qacEΔ1* or both variants of the gene and the group that did not harbour either the gene or its variant. Tolerance or resistance to this disinfectant is likely not imparted by the action of efflux pumps as the mechanism of action of NaOCl is independent of the influx of NaOCl into bacterial cells. Rather, NaOCl induces a stress response resulting in increased frequency of horizontal gene transfer [28]. Moreover, it is known to induce saponification, amino acid neutralization and chloramination which interfere with cellular metabolic activity and eventually cause cell death [35]. In addition, oxidative stress imparted by the activity of NaOCl has been demonstrated to downregulate formation of biofilms in *K. pneumoniae* by disrupting the expression of type 3 fimbriae crucial for biofilm formation [36]. Sodium hypochlorite has been shown to clear biofilms in *K. pneumoniae* [37], and although more resistant to chlorine-based disinfectants, biofilms of *E. cloacae* have also been shown to be highly sensitive to chlorine-releasing disinfectants such as NaOCl [38]. Likely, induction of the bacterial stress response when challenged with hypochlorous acid offers minimal protection against sodium hypochlorite.

Interestingly, in a study performed by Chen et al. [39] over a 60-day period, exposure of *K. pneumoniae* to NaOCl induced genetic mutations that affect pathogenicity, tolerance to NaOCl, biofilm formation and cross-resistance to several antibiotics. Furthermore, Guo et al. [31] found an association between carriage of *qacE* and *qacEΔ1* with resistance to ‘84’ (a chlorine-releasing agent) which contains NaOCl. These findings highlight the complex interplay between disinfectant exposure, bacterial physiology and the development of resistance mechanisms, underscoring the ongoing need for further enquiry in this area to better understand and mitigate the emergence of biocide tolerance/resistance to NaOCl.

It is important to note that the MIC_50_, MIC_90_ and mean MBC of NaOCl for isolates in this study ranged between 7031 mg/L and 9375 mg/L, 14,060 mg/L and 18,750 mg/L and 48,750 mg/L and 56,250 mg/L, respectively, (Table 1) after 24 h exposure, which are higher than 5000 mg/L that is used for the disinfection of benches in Princess Marina Hospital and other hospitals in Botswana. To achieve rapid killing of bacteria, disinfectants are usually used in concentrations much higher than their MBCs, as this prevents survival and development of resistance. Therefore, results of the current study may indicate increasing tolerance because of sub-lethal exposure, taking into consideration the extensive use of NaOCl for years, in the hospital environment [39]. As the experimental set up did not consider the types of surfaces, dust and debris and exposure time, the MIC and MBC for these isolates may be higher than 18,750 mg/L and 56,250 mg/L, respectively.

Analysis of individual isolates revealed a large variation in susceptibility to NaOCl. The MIC ranged from 2300 mg/L to 37,500 mg/L, while MBC spanned from 9400 mg/L to greater than 75,000 mg/L. This wide range may be partially attributed to the limitations of the two-fold microbroth serial dilution method employed. This method offers limited resolution with low accuracy, and therefore, the MICs of individual isolates may encompass a large concentration spectrum. Furthermore, it is important to consider that NaOCl is typically used in combination with 70% alcohol for disinfection. Therefore, the MIC and MBC values determined in this study should only be interpreted as a guide.

A major limitation in this study is that other determinants of biocide resistance, such as biofilm formation, were not evaluated. Consequently, the interpretation of biocide resistance is restricted to only one of many potential contributing factors. In addition, only antimicrobial drug-resistant strains were included in this study. Incorporating both drug-sensitive and drug-resistant bacteria would have facilitated a comprehensive comparison of *qacE* genetic variation and MIC/MBC distribution. Furthermore, the antimicrobial resistance phenotypes were determined through presumptive screening, based on concordant results obtained from selective chromogenic media and VITEK-2.

Nevertheless, the current study provides insights on the biocide resistance in the context of sodium hypochlorite and serves as a foundation for future biocide resistance research in Botswana.

## 4. Methods

### 4.1. Selection of Bacterial Strains, Identification, and Antimicrobial Susceptibility Testing

Rectal and skin swabs were collected from patients admitted to Princess Marina Hospital in Gaborone, Botswana, who were recruited into the Multi Drug Resistant Organisms (MDROs) Colonization and Intensive Care Unit/Neonatal Unit (ICU/NNU) point prevalence survey studies, conducted by the Surveillance of Healthcare Associated infections and Antimicrobial Resistance (SHARE) program [40]. The collection period spanned from June 2022 to May 2023 across various wards in Princess Marina Hospital. The samples were collected using flocked swabs and transported in Eswab^®^ (COPAN, Brescia, Italy) transport media. Upon reception at the laboratory, the swabs were directly inoculated on selective and differential chromogenic media, specifically CHROMAgar ESBL and CHROMAgar CRE (CHROMAgar, Saint-Denis, France), for preliminary identification of colonies belonging to the KESC group (*Klebsiella/Enterobacter/Serratia/Citrobacter*) [15]. The media were incubated aerobically at 37 °C for 16–24 h. Colonies producing a blue colour on the media were presumptively identified as belonging to the KESC group. To ensure purity, the presumptive KESC colonies were sub-cultured on nutrient agar. Colony identification and antimicrobial susceptibility testing (AST) were conducted using the VITEK-2 Compact (bioMérieux, Marcy-l’Étoile, France) automated microbial identification system. VITEK-2 GN (Gram-negative) and VITEK-2 AST-N256 cards were employed for colony identification and AST, respectively. The VITEK-2 uses an “advanced expert system” to presumptively determine ESBL and CARBA phenotypes by comparing each MIC value against a database of phenotypes and MIC distributions to infer resistance mechanisms [41]. Antibiotic MICs results were interpreted following the clinical breakpoints as reported in the 30th edition of the CLSI standards [27]. All *K. pneumoniae*, *K. oxytoca, K. aerogenes* and *E. cloacae* were retained for further analysis. This included molecular genotyping (PCR) for the gene of interest and determination of Minimum Inhibitory Concentration (MIC) and Minimum Bactericidal Concentration (MBC) of sodium hypochlorite.

### 4.2. Minimum Inhibitory Concentration (MIC) and Minimum Bactericidal Concentration (MBC) of Biocides

The MIC of sodium hypochlorite (NaOCl) was determined using the Resazurin microtiter method [42]. Chemical-grade sodium hypochlorite solution (Rochelle Chemicals, South Africa), with concentration of 15% (150 g available chlorine per litre), was used for serial dilutions in the MIC determination. The use of Resazurin for MIC determination is a standardized microbroth dilution method which relies on the redox reaction imparted by metabolically active bacterial cells on Resazurin. Active metabolic cells reduce Resazurin (blue) into Resorufin (pink), which can be translated into a quantifiable measure of bacterial activity in a certain concentration of biocide [28]. Sodium hypochlorite was two-fold serially diluted in Mueller–Hinton broth using round-bottom sterile 96-well microplates to achieve a concentration range between 0.015% and 7.5% (150 mg/L and 75,000 mg/L). Overnight Mueller–Hinton broth cultures of the test organisms grown at 37 °C were diluted to 0.5 McFarland turbidity standard. To achieve a recommended inoculum of 5 × 10^5^ CFU/mL, as per EUCAST guidelines on broth microdilution susceptibility testing [43], 25 µL of the fresh 0.5 MacFarland standard bacterial culture was inoculated into 5 mL of nutrient broth. Fifty (50) µL of this bacterial suspension was inoculated into the prepared 96 well plates. A positive control (Mueller–Hinton broth and bacterial cells) and negative controls (Mueller–Hinton broth only) were included in the microtitreplate. After a 24 h incubation, 50 µL of 0.1% Resazurin was added to all wells followed by an incubation of 4 h at 37 °C, after which colour change was observed. Wells were assessed visually: a colour change from blue to pink was taken as indication of bacterial growth, while the well with the minimum concentration of biocide in which there is no observable reduction in Resazurin was determined to be MIC. The MIC experiments were performed in duplicates. After MIC determination, all wells with no observable reduction in Resazurin, were cultured onto nutrient agar for 24 h at 37 °C. The lowest concentration in which there was no bacterial growth was determined to be MBC. The MBC was defined as the lowest concentration of biocide which kills 99.9% of bacterial cells or no growth. *Escherichia coli* ATCC 25922 and *K. pneumoniae* BAA-1705 were used as control strains for the MIC and MBC experiments.

### 4.3. DNA Extraction, PCR and Restriction Digest

DNA was extracted from pure cultures using the Zymogen Quick-DNA Miniprep kit (Zymo Research, Irvine, CA, USA). Primers for *qacE* and *qacE∆1* were adapted from Kazama et al. [22]. However, the sequence for the *qacE∆1* reverse primer given in this publication was incorrect. It was written in the 3′ to 5′ direction. The corrected sequence is given below. Primers were synthesized by Inqaba biotechnical Industries (Pretoria, South Africa). The primer sequences were as follows:

Common forward-*qacE-F/qacE∆1*-F: GCCCTACACAAATTGGGAGA

Deletion reverse-*qacE∆1*-R: AACACCGTCACCATGGCGTC

Wildtype reverse-*qacE*-R: TTAGTGGGCACTTGCTTTGG

For PCR, we used 12.5 μL OneTaq quickload 2X Mastermix with standard buffer (New England Biolabs, Ipswich, MA, USA) with 9.5 μL of ddH_2_O, 0.5 μL (0.2 μMol) of each primer and 2 μL of extracted DNA in a 25 μL reaction. PCR was performed in 30 cycles of steps as follows: denaturation at 94° for 1 min, annealing for *qacE* at 55 °C, and *qacE∆1* at 51 °C for 30 s and extension at 68 °C for 1 min. Moreover, we analysed the sequences of the *qacE* and *qacE∆1* genes from Kazama et al. [22] to identify priming sites, start and stop codons, the portion of the sequence that is similar in both genes and a unique *HinfI* restriction site. To confirm the identity of each PCR product, a restriction digest was performed on both PCR products using the enzyme *HinfI* (New England Biolabs, Ipswich, MA, USA)**.** After PCR amplification, 10 μL of each PCR product was digested with 1 μL of *HinfI* (New England Biolabs, USA). Digestion reactions were carried out in the NEB buffer at 37 °C for 1 h. Digestion products were analysed on a 2% agarose gel.

### 4.4. Statistical Analysis

After characterizing the data with descriptive statistics, regression analysis was used to explore any potential association between *qacE* genotypes, antimicrobial drug resistance and MIC and MBC of sodium hypochlorite for all isolates. Comparison of MIC_50_ of sodium hypochlorite and mean MBC in isolates carrying [*qacE* and/or *qacEΔ1*] vs. [no gene] were performed using the Mann–Whitney U test and Student’s *t*-test, respectively. The IBM SPSS Statistics software (version 29.0.2.0) was used for the analysis.

### 4.5. Biosafety

The study adhered to standard biosafety protocols, mandating the use of a laboratory coat, gloves and eye protection. All manipulations involving bacterial cultures were performed within a biological safety cabinet (BSC) at the University of Botswana.

### 4.6. Ethical Approval

Ethical approval was sought and obtained from the University of Botswana Institutional Review Board (UBR/RES/IRB/BIO/GRAD/329), the Botswana Ministry of Health and Wellness Health Research and Development Division (HPRD/6/14/1) and Princess Marina Hospital Institutional Review Board (PMH 2/11AII/164).

## 5. Conclusions

There is high prevalence of *qacEΔ1* in clinical isolates of *K. pneumoniae, K. oxytoca*, *K. aerogenes* and *E. cloacae* collected from Princess Marina Hospital in Gaborone, Botswana. Although the MIC of NaOCl is higher than that used for bench disinfection in hospitals, these genes do not seem to be associated with tolerance or resistance to sodium hypochlorite in the context of this study and are also not associated with ESBL or carbapenem resistance. This calls for a review on the application of NaOCl and corresponding increments in the working concentrations of this biocide and other chlorine-releasing agents in this context.

## Figures and Tables

**Table 1:** MIC_50_, MIC_90_ and mean MBC of sodium hypochlorite (NaOCl) by bacterial species.

Bacterial Species (N)	MIC_50_ (mg/L)	MIC_90_ (mg/L)	Mean MBC (mg/L)
*K. pneumoniae* (143)	9375	18,750	55,460
*K. aerogenes* (26)	9375	18,750	56,250
*K. oxytoca* (5)	7031	14,060	48,750
*E. cloacae* (42)	9375	18,750	54,240

**Table 2:** Frequency of *qacE* and *qacEΔ1* in clinical isolates of *K. pneumoniae*, *K. oxytoca*, *K. aerogenes* and *E. cloacae*.

Bacterial Species (N)	*qacE* N (%; 95% CI)	*qacEΔ1* N (%; 95% CI)
*K. pneumoniae* (143)	21 (14.69; 9.33–21.57)	114 (79.72; 72.19–85.98)
*E. cloacae* (42)	8 (19.05; 8.60–34.12)	31 (73.81; 57.96–86.14)
*K. aerogenes* (26)	3 (11.54; 2.45–30.15)	23 (88.46; 69.85–97.55)
*K. oxytoca* (5)	1 (20.00; 0.51–71.64)	5 (100; 39.76–100.00)
Total (216)	33 (15.28; 10.76–20.78)	174 (80.56; 74.64–85.61)

## Data Availability

Data available on request due to restrictions (ethical reasons).

## Supplementary Materials

The following supporting information can be downloaded at: https://www.mdpi.com/article/10.3390/antibiotics14070662/s1, Table S1: Cephalosporin and carbapenem MICs (µg/mL) and antimicrobial susceptibility phenotypes.
