# Supplementary material for: Lack of Association Between qacE and qacE∆1 Gene Variants and Sodium Hypochlorite Resistance in Clinical Isolates of ESBL- and Carbapenemase-Producing Klebsiella spp. and Enterobacter spp., from Gaborone, Botswana
Source: Antibiotics (Basel). 2025 Jun 30;14(7):662. doi: 10.3390/antibiotics14070662 (PMC12291931; doi:10.3390/antibiotics14070662)
Supplement: Supplementary file 1 [file antibiotics-14-00662-s001.zip › antibiotics-3542670-supplementary.pdf]

Isolate (species)	AST	Cefuroxime axetil	Cefoxitin	Cefotaxime	Cefepime	Ertapenem	Meropenem	Ceftazidime	Imipenem
K. aerogenes	ESBL	≥64	≥64	≥64	32	≤0.5	≤0.25	16	≤0.25
K. pneumoniae	ESBL	≥64	8	≥64	≥64	≤0.5	≤0.25	4	≤0.25
K. pneumoniae	ESBL	≥64	≥4	≥64	≥64	≤0.5	≤0.25	≥64	≤0.25
K. pneumoniae	ESBL	≥64	32	≥64	2	2	≤0.25	32	≤0.25
K. pneumoniae	ESBL	≥64	≥64	≥64	4	2	≤0.25	≥64	≤0.25
K. pneumoniae	ESBL	≥64	8	≥64	32	≤0.5	≤0.25	≥64	0.5
K. pneumoniae	ESBL	≥64	≥4	≥64	2	≤0.5	≤0.25	32	≤0.25
K. pneumoniae	ESBL	≥64	≥64	≥64	≥64	≥8	≥16	≥64	≥16
K. pneumoniae	ESBL, CARBA	≥64	≥64	≥64	≥64	≥8	≥16	≥64	≥16
K. pneumoniae	CARBA	≥64	≥64	≥64	16	≥8	≥16	≥64	≥16
E. cloacae	CARBA	4	N/A	≤1	≤1	≥8	≥16	≤1	≥16
K. pneumoniae	ESBL	≥64	16	≥64	32	≤0.5	≤0.25	≥64	≤0.25
K. oxytoca	CARBA	≥64	≥64	≥64	≥64	≥8	≥16	≥64	≥16
K. pneumoniae	ESBL	≥64	≤4	16	4	≤0.5	≤0.25	≤1	≤0.25
K. pneumoniae	CARBA	≥64	≤4	16	4	≤0.5	4	≤1	2
K. pneumoniae	CARBA	≥64	≥64	≥64	≥64	≥8	≥16	≥64	≥16

K. pneumoniae	ESBL	≥64	≤4	16	4	≤0.5	≤0.25	≤1	≤0.25
K. pneumoniae	ESBL	≥64	≤4	≥64	32	≤0.5	≤0.25	≥64	≤0.25
K. aerogenes	ESBL	≥64	≤4	≥64	2	≤0.5	≤0.25	8	≤0.25
E. cloacae	ESBL	≥64	N/A	≥64	32	≤0.5	≤0.25	16	0.5
K. aerogenes	CARBA	≥64	≥64	≥64	≥64	≥8	≥16	≥64	≥16
K. pneumoniae	CARBA	≥64	≥64	≥64	≥64	≥8	≥16	≥64	≥16
K. pneumoniae	ESBL	≥64	≥64	16	≤1	≤0.5	≤0.25	≥64	1
E. cloacae	ESBL	≥64	N/A	≥64	2	≤0.5	≤0.25	8	≤0.25
K. aerogenes	ESBL	≥64	≥4	≥64	32	≤0.5	≤0.25	16	0.5
E. cloacae	ESBL	≥64	N/A	≥64	≤1	≤0.5	≤0.25	≥64	0.5
E. cloacae	ESBL	≥64	N/A	≥64	2	4	≤0.25	≥64	≤0.25
K. pneumoniae	ESBL	≥64	≤4	≥64	2	≤0.5	≤0.25	4	≤0.25
E. cloacae	ESBL	≥64	N/A	≥64	2	≤0.5	≤0.25	8	≤0.25
K. pneumoniae	ESBL	≥64	≥4	≥64	32	≤0.5	≤0.25	16	0.5
E. cloacae	ESBL	≥64	N/A	≥64	≥64	4	≤0.25	≥64	≤0.25
K. pneumoniae	CARBA	4	≤1	≤1	≤1	4	4	≤1	2
K. aerogenes	ESBL	≥64	≥64	≥64	≥64	≤0.5	≤0.25	≥64	0.5

K. pneumoniae	ESBL	≥64	≤4	≥64	≥64	≤0.5	≤0.25	16	≤0.25
K. pneumoniae	CARBA	≥64	≤4	8	1	≤0.5	≤0.25	4	≤0.25
E. cloacae	ESBL	≥64	N/A	≥64	4	≤0.5	≤0.25	≥64	0.5
K. pneumoniae	CARBA	≥64	≥64	≥64	≤1	≤0.5	≤0.25	≥64	≤0.25
E. cloacae	CARBA	≥64	N/A	≥64	4	≥8	8	≥64	2
E. cloacae	ESBL	≥64	N/A	≥64	2	≤0.5	≤0.25	16	≤0.25
E. cloacae	ESBL	≥64	N/A	8	≤1	≤0.5	≤0.25	2	≤0.25
K. pneumoniae	ESBL	≥64	32	≥64	≤1	≤0.5	≤0.25	≥64	≤0.25
K. pneumoniae	ESBL	≥64	≥4	≥64	≥64	≤0.5	≤0.25	≥64	≤0.25
K. pneumoniae	ESBL	≥64	≤4	≥64	2	≤0.5	≤0.25	8	≤0.25
E. cloacae	CARBA	≥64	N/A	≥64	4	≤0.5	≤0.25	16	≤0.25
E. cloacae	CARBA	≥64	N/A	≥64	2	≤0.5	≤0.25	4	≤0.25
K. pneumoniae	CARBA	≥64	32	≥64	≥64	≥8	2	≥64	0.5
K. pneumoniae	CARBA	≥64	≥64	≥64	≥64	≥8	1	≥64	0.5
K. pneumoniae	CARBA	≤4	≤1	≤1	≤1	≤0.5	2	≤1	2
K. pneumoniae	CARBA	4	≤1	≤1	≤1	≤0.5	4	≤1	2
K. pneumoniae	CARBA	≥64	≥64	≤1	≤1	≤0.5	2	≤1	0.5

K. pneumoniae	ESBL	≥64	≥64	≥64	32	≤0.5	≤0.25	16	≤0.25
K. pneumoniae	ESBL	≥64	8	≥64	≥64	≤0.5	≤0.25	4	≤0.25
K. pneumoniae	CARBA	≥64	≥4	≥64	≥64	≤0.5	≤0.25	≥64	≤0.25
K. pneumoniae	CARBA	≥64	32	≥64	2	2	≤0.25	32	≤0.25
K. pneumoniae	ESBL	≥64	≤4	≥64	≥64	≤0.5	≤0.25	16	≤0.25
K. pneumoniae	ESBL	≥64	≤4	≥64	2	≤0.5	≤0.25	16	≤0.25
K. pneumoniae	CARBA	≥64	32	≥64	32	≥8	8	≥64	8
E. cloacae	ESBL	≥64	N/A	≥64	≤1	≤0.5	≤0.25	≥64	≤0.25
K. aerogenes	CARBA	≥64	≥64	≥64	≤1	2	≤0.25	≥64	1
K. pneumoniae	ESBL	≥64	≥4	≥64	2	≤0.5	≤0.25	16	≤0.25
E. cloacae	ESBL	≥64	N/A	≥64	≥64	≤0.5	≤0.25	16	≤0.25
E. cloacae	ESBL	≥64	N/A	≥64	2	≤0.5	≤0.25	8	≤0.25
K. pneumoniae	ESBL	≥64	≤4	≥64	2	≤0.5	≤0.25	4	≤0.25
E. cloacae	ESBL	≥64	N/A	≥64	≥64	≤0.5	≤0.25	≥64	≤0.25
E. cloacae	ESBL	≥64	N/A	≥64	≥64	≤0.5	≤0.25	≥64	≤0.25
E. cloacae	ESBL	≥64	N/A	≥64	≥64	≤0.5	≤0.25	≥64	≤0.25
K. aerogenes	ESBL	≥64	≤4	≥64	2	≤0.5	≤0.25	4	≤0.25

E. cloacae	CARBA	≥64	N/A	≥64	≥64	≥8	4	≥64	1
K. pneumoniae	ESBL	16	≤4	8	≤1	≤0.5	≤0.25	16	≤0.25
E. cloacae	ESBL	≥64	N/A	32	≤1	≤0.5	≤0.25	16	≤0.25
K. pneumoniae	ESBL	≥64	≤4	≥64	2	≤0.5	≤0.25	4	≤0.25
E. cloacae	ESBL	≥64	N/A	≥64	≤1	≤0.5	≤0.25	≥64	≤0.25
E. cloacae	ESBL	≥64	N/A	≥64	2	≤0.5	4	≥64	≤0.25
K. pneumoniae	ESBL	≥64	≤4	≥64	2	≤0.5	≤0.25	8	0.5
K. oxytoca	ESBL	8	≤4	≤1	≤1	≤0.5	≤0.25	16	≤0.25
K. pneumoniae	ESBL	≥64	≤4	≥64	2	≤0.5	≤0.25	8	≤0.25
K. pneumoniae	ESBL	≥64	≤4	≥64	4	≤0.5	≤0.25	16	≤0.25
K. pneumoniae	ESBL	≥64	≤4	≥64	2	≤0.5	≤0.25	4	≤0.25
K. pneumoniae	ESBL	≥64	≤4	≥64	≤1	≤0.5	≤0.25	≤1	≤0.25
K. pneumoniae	ESBL	≥64	≤4	≥64	2	≤0.5	≤0.25	8	≤0.25
K. pneumoniae	ESBL	≥64	≤4	≥64	≤1	≤0.5	≤0.25	≥64	≤0.25
K. aerogenes	CARBA	≥64	32	≥64	≥64	≥8	8	≥64	8
K. pneumoniae	ESBL	≥64	≤4	≥64	2	≤0.5	≤0.25	4	≤0.25
K. pneumoniae	ESBL	≥64	≤4	≥64	2	≤0.5	≤0.25	4	≤0.25

K. pneumoniae	ESBL	≥64	≤4	≥64	2	≤0.5	≤0.25	4	≤0.25
K. pneumoniae	ESBL	≥64	≤4	≥64	≥64	≤0.5	≤0.25	8	≤0.25
K. pneumoniae	ESBL	≥64	≤4	≥64	2	≤0.5	≤0.25	8	≤0.25
K. aerogenes	CARBA	≥64	≤4	≥64	≥64	≤0.5	≤0.25	8	≤0.25
K. pneumoniae	ESBL	≥64	8	≥64	2	4	≤0.25	4	≤0.25
E. cloacae	ESBL	≥64	N/A	≥64	≥64	≤0.5	≤0.25	8	≤0.25
K. oxytoca	ESBL	≥64	8	≥64	≥64	≤0.5	≤0.25	≥64	≤0.25
K. pneumoniae	ESBL	≥64	≤4	≥64	4	≤0.5	≤0.25	8	≤0.25
K. pneumoniae	ESBL	≥64	≤4	≥64	2	≤0.5	≤0.25	8	≤0.25
K. pneumoniae	ESBL	≥64	≤4	≥64	≤1	≤0.5	≤0.25	≥64	≤0.25
K. pneumoniae	ESBL	≥64	≤4	≥64	≥64	≤0.5	≤0.25	16	≤0.25
K. pneumoniae	ESBL	≥64	≤4	16	≤1	≤0.5	≤0.25	≤1	≤0.25
K. pneumoniae	ESBL	≥64	≤4	≥64	2	≤0.5	≤0.25	≥64	≤0.25
K. pneumoniae	ESBL	≥64	≤4	≥64	2	≤0.5	≤0.25	4	≤0.25
K. aerogenes	ESBL	≥64	≤4	≥64	≥64	≤0.5	≤0.25	≥64	≤0.25
K. pneumoniae	ESBL	≥64	≤4	≥64	≥64	≤0.5	≤0.25	16	≤0.25
K. pneumoniae	ESBL	≥64	≤4	≥64	2	≤0.5	≤0.25	16	≤0.25

K. aerogenes	ESBL	≥64	8	≥64	2	≤0.5	≤0.25	16	≤0.25
K. pneumoniae	ESBL	≥64	≤4	8	≤1	≤0.5	≤0.25	2	≤0.25
E. cloacae	ESBL	≥64	N/A	≥64	≤1	≤0.5	≤0.25	≥64	≤0.25
K. pneumoniae	ESBL	≥64	≤4	≥64	≥64	≤0.5	≤0.25	16	≤0.25
K. pneumoniae	ESBL	≥64	≤4	≥64	≥64	≤0.5	≤0.25	16	≤0.25
K. pneumoniae	ESBL	4	4	≤1	≤1	≤0.5	≤0.25	≤1	≤0.5
K. aerogenes	CARBA	≥64	≥64	≥64	8	≥8	≥16	≥64	≥16
K. pneumoniae	CARBA	≥64	≥64	≥64	≥64	≥8	≥16	≥64	≥16
E. cloacae	ESBL	≥64	N/A	32	2	≤0.5	≤0.25	2	≤0.25
K. aerogenes	ESBL	≥64	≤4	≥64	≥64	≤0.5	≤0.25	≥64	≤0.25
K. aerogenes	ESBL	4	≤4	≤1	≤1	≤0.5	≤0.25	≤1	≤0.25
K. pneumoniae	ESBL	≥64	≥64	≥64	32	≤0.5	≤0.25	16	≤0.25
K. aerogenes	ESBL	4	≥64	≤1	≤1	≤0.5	≤0.25	≤1	≤0.5
K. oxytoca	ESBL	≥64	≥64	≤4	≤1	≤1	≤0.25	≥64	≤0.5
E. cloacae	ESBL	2	N/A	≤1	≤1	≤0.5	≤0.25	≤1	≤0.5
K. aerogenes	CARBA	≥64	≥64	≥64	8	≥8	≥16	≥64	≥16
K. pneumoniae	ESBL	≥64	≤4	≥64	≥64	≤0.5	≤0.25	≥64	≤0.25

K. pneumoniae	ESBL	≥64	≥64	≥64	8	≥8	≥16	≥64	≥16
K. pneumoniae	ESBL	≥64	≤4	≥64	4	≤0.5	≤0.25	16	≤0.25
K. pneumoniae	ESBL	≥64	≤4	≥64	32	≤0.5	≤0.25	4	≤0.25
K. pneumoniae	ESBL	≥64	≥64	≥64	32	≤0.5	≤0.25	16	≤0.25
E. cloacae	ESBL	≥64	N/A	≥64	4	≤0.5	≤0.25	16	≤0.25
K. aerogenes	ESBL	≥64	≤4	≥64	≥64	≤0.5	≤0.25	16	≤0.25
K. pneumoniae	ESBL	≥64	≤4	≥64	≥64	≤0.5	≤0.25	16	≤0.25
K. pneumoniae	ESBL	≥64	≤4	≥64	2	≤0.5	≤0.25	16	≤0.25
K. pneumoniae	ESBL	≥64	≤4	≥64	2	≤0.5	≤0.25	8	≤0.25
K. pneumoniae	ESBL	≥64	≤4	≥64	≤1	≤0.5	≤0.25	≤1	≤0.25
K. pneumoniae	ESBL	≥64	≤4	≥64	≤1	≤0.5	≤0.25	≤1	≤0.25
K. pneumoniae	ESBL	≥64	≥64	≥64	2	≤0.5	≤0.25	≤1	≤0.25
K. pneumoniae	ESBL	≥64	≥64	≥64	2	≤0.5	≤0.25	≤1	≤0.25
E. cloacae	ESBL	≥64	N/A	≥64	2	≤0.5	≤0.25	≥64	≤0.25
K. pneumoniae	ESBL	≥64	≥4	≥64	4	≤0.5	≤0.25	≥64	≤0.25
K. pneumoniae	ESBL	≥64	≥4	≥64	≤1	≤0.5	≤0.25	≤1	≤0.25
E. cloacae	ESBL	≥64	N/A	≥64	≤1	≤0.5	≤0.25	≥64	≤0.25

K. pneumoniae	ESBL	≥64	≥4	≥64	2	≤0.5	≤0.25	16	≤0.25
K. pneumoniae	ESBL	≥64	4	≥64	≥64	≤0.5	≤0.25	8	≤0.25
E. cloacae	ESBL	≥64	N/A	≥64	≥64	≤0.5	≤0.25	8	≤0.25
K. pneumoniae	ESBL	≥64	≥4	≥64	4	≤0.5	≤0.25	16	≤0.25
K. aerogenes	ESBL	≥64	8	≥64	32	≤0.5	≤0.25	≥64	2
K. pneumoniae	ESBL	≥64	≥4	≥64	4	≤0.5	≤0.25	16	≤0.25
E. cloacae	CARBA	≥64	N/A	≥64	≥64	≥8	4	≥64	2
E. cloacae	ESBL	≥64	N/A	≥64	2	≤0.5	≤0.25	≥64	≤0.25
E. cloacae	ESBL	≥64	N/A	≥64	2	4	≤0.25	≥64	1
K. oxytoca	ESBL	≥64	≤4	≥64	2	≤0.5	≤0.25	8	≤0.25
K. pneumoniae	ESBL	≥64	≤4	≥64	4	≤0.5	≤0.25	16	≤0.25
E. cloacae	ESBL	≥64	N/A	≥64	≤1	≤0.5	≤0.25	≥64	≤0.25
E. cloacae	CARBA	≥64	N/A	≥64	≥64	≥8	8	≥64	1
K. aerogenes	ESBL	≥64	≤4	≥64	≤1	≤0.5	≤0.25	8	≤0.25
K. pneumoniae	ESBL	≥64	≤4	2	≤1	≤0.5	≤0.25	≥64	≤0.25
K. aerogenes	ESBL	≥64	≤4	2	≤1	≤0.5	≤0.25	≥64	≤0.25
K. pneumoniae	ESBL	≥64	≤4	≥64	2	≤0.5	≤0.25	8	≤0.25

K. pneumoniae	ESBL	≥64	≤4	≥64	2	≤0.5	≤0.25	4	≤0.25
K. aerogenes	ESBL	≥64	≤4	≥64	2	≤0.5	≤0.25	4	≤0.25
K. pneumoniae	ESBL	≥64	≤4	≥64	≥64	≤0.5	≤0.25	16	≤0.25
K. pneumoniae	ESBL	≥64	≤4	≥64	4	≤0.5	≤0.25	4	≤0.25
E. cloacae	ESBL	≥64	N/A	≥64	≤1	≤0.5	≤0.25	≥64	≤0.25
K. aerogenes	ESBL	≥64	≥64	≥64	4	≤0.5	≤0.25	≥64	≤0.25
K. pneumoniae	ESBL	≥64	≤4	≥64	≥64	≤0.5	≤0.25	≥64	≤0.25
K. pneumoniae	ESBL	≥64	≤4	≥64	2	≤0.5	≤0.25	4	≤0.25
K. pneumoniae	ESBL	≥64	≤4	≥64	≥64	≤0.5	≤0.25	≥64	≤0.25
K. pneumoniae	ESBL	≥64	≤4	≥64	2	≤0.5	≤0.25	8	≤0.25
K. pneumoniae	ESBL	≥64	≤4	≥64	2	≤0.5	≤0.25	4	≤0.25
K. pneumoniae	ESBL	≥64	≤4	≥64	4	≤0.5	≤0.25	≥64	≤0.25
K. pneumoniae	ESBL	≥64	≤4	≥64	4	≤0.5	≤0.25	≥64	≤0.25
K. aerogenes	ESBL	≥64	8	≥64	≥64	≤0.5	≤0.25	≥64	≤0.25
K. pneumoniae	ESBL	≥64	≤4	≥64	32	≤0.5	≤0.25	≥64	≤0.25
K. pneumoniae	CARBA	≥64	8	≥64	≥64	≤0.5	≤0.25	≥64	≤0.25
K. pneumoniae	ESBL	≥64	≤4	≥64	2	≤0.5	≤0.25	≥64	≤0.25

K. pneumoniae	ESBL	≥64	≥4	≥64	4	≤0.5	≤0.25	8	≤0.25
K. pneumoniae	ESBL	≥64	≤4	≥64	≤1	≤0.5	≤0.25	4	≤0.25
K. aerogenes	ESBL	≥64	≤4	≥64	≤1	≤0.5	≤0.25	4	≤0.25
E. cloacae	ESBL	≥64	N/A	≥64	4	≤0.5	≤0.25	16	≤0.25
K. pneumoniae	ESBL	≥64	≥4	≥64	4	≤0.5	≤0.25	≥64	≤0.25
K. pneumoniae	ESBL	≥64	≥4	≥64	4	≤0.5	≤0.25	8	≤0.25
K. pneumoniae	ESBL	≥64	≥4	≥64	8	≤0.5	≤0.25	4	≤0.25
K. pneumoniae	ESBL	≥64	≥4	≥64	8	≤0.5	≤0.25	4	≤0.25
K. pneumoniae	ESBL	≥64	≤4	≥64	2	≤0.5	≤0.25	8	≤0.25
K. pneumoniae	ESBL	≥64	≤4	≥64	2	≤0.5	≤0.25	4	≤0.25
K. pneumoniae	ESBL	≥64	≥4	≥64	8	≤0.5	≤0.25	≥64	≤0.25
K. pneumoniae	CARBA	≥64	32	≥64	32	≥8	8	≥64	8
K. aerogenes	ESBL	≥64	≤4	≥64	4	≤0.5	≤0.25	16	≤0.25
K. pneumoniae	ESBL	≥64	≥64	≥64	≥64	≤0.5	≤0.25	16	≤0.25
E. cloacae	ESBL	≥64	N/A	≥64	≥64	≤0.5	≤0.25	16	≤0.25
E. cloacae	ESBL	≥64	N/A	≥64	≥64	≤0.5	≤0.25	16	≤0.25
K. pneumoniae	ESBL	≥64	≤4	≥64	2	≤0.5	≤0.25	16	≤0.25

K. pneumoniae	CARBA, ESBL	≥64	≥64	≥64	≥64	≥8	≥16	≥64	8
K. pneumoniae	ESBL	≥64	≥4	≥64	4	≤0.5	≤0.25	16	≤0.25
E. cloacae	ESBL	≥64	N/A	≥64	4	4	≤0.25	≥64	≤0.25
K. pneumoniae	ESBL	≥64	32	≥64	≥64	≤0.5	8	≥64	0.5
K. pneumoniae	ESBL	≥64	16	≥64	≥64	≤0.5	≤0.25	≥64	≤0.25
K. aerogenes	ESBL	≥64	≥64	≥64	≥64	1	≤0.25	≥64	≤0.25
K. pneumoniae	ESBL	≥64	≤4	≥64	2	≤0.5	≤0.25	16	≤0.25
E. cloacae	CARBA	≥64	N/A	≥64	32	≥8	≥16	≥64	8
K. pneumoniae	ESBL	≥64	≥64	≥64	8	≤0.5	≤0.25	16	≤0.25
K. pneumoniae	CARBA	≥64	≥64	≥64	≥64	≥8	≥16	≥64	8
K. pneumoniae	CARBA	≥64	≥64	≥64	≥64	≥8	1	≥64	≤0.25
K. pneumoniae	CARBA	≥64	≥64	≥64	≥64	≥8	1	≥64	≤0.25
K. pneumoniae	CARBA	≥64	≤4	≥64	32	≤0.5	≤0.25	≥64	≤0.25
K. pneumoniae	ESBL	≥64	≤4	≥64	32	≤0.5	≤0.25	≥64	≤0.25
K. pneumoniae	ESBL	≥64	≤4	≥64	4	≤0.5	≤0.25	≥64	≤0.25
K. pneumoniae	ESBL	≥64	≥4	≥64	2	≤0.5	≤0.25	4	≤0.25
K. pneumoniae	ESBL	≥64	16	≥64	≥64	≤0.5	≤0.25	≥64	≤0.25

K. pneumoniae	ESBL	≥64	≤4	≥64	≤1	≤0.5	≤0.25	≥64	≤0.25
K. pneumoniae	ESBL	≥64	≤4	≥64	32	≤0.5	≤0.25	≥64	≤0.25
K. pneumoniae	ESBL	≥64	≥64	≥64	≥64	≤0.5	≤0.25	≥64	≤0.25
K. pneumoniae	ESBL	≥64	≥4	≥64	≥64	≤0.5	≤0.25	≥64	≤0.25
K. pneumoniae	ESBL	≥64	≥4	≥64	4	≤0.5	≤0.25	8	≤0.25
K. pneumoniae	ESBL	≥64	≥4	≥64	≥64	≤0.5	≤0.25	≥64	≤0.25
K. pneumoniae	ESBL	≥64	≥4	≥64	≥64	≤0.5	≤0.25	≥64	≤0.25
K. pneumoniae	ESBL	≥64	16	8	≤1	≤0.5	1	16	≤0.25
K. pneumoniae	ESBL	≥64	≤4	≥64	4	≤0.5	≤0.25	32	≤0.25
K. pneumoniae	ESBL	≥64	≤4	≥64	32	≤0.5	≤0.25	≥64	≤0.25
K. pneumoniae	ESBL	≥64	≤4	≥64	4	≤0.5	≤0.25	≥64	≤0.25
K. pneumoniae	ESBL	≥64	≤4	16	2	≤0.5	≤0.25	4	≤0.25
K. pneumoniae	CARBA	≥64	≥64	≥64	32	≥8	8	≥64	8

Supplementary table S1. Cephalosporin and carbapenem MICs (µg/ml) and antimicrobial susceptibility phenotypes.
